# O1-7 MoveHealthy: improving health and sustaining participation of youth in sports through sports injury prevention

**DOI:** 10.1093/eurpub/ckac094.007

**Published:** 2022-08-29

**Authors:** Johan de Jong

**Affiliations:** School of Sportstudies, Hanzehogeschool Groningen, Groningen, The Netherlands

**Keywords:** Injury prevention, mapping, physical education, sports

## Abstract

**Background:**

Sport injuries are a major reason for reduced participation and drop-out from sports and physical education. Refraining from sport participation has negative effects on mental and physical well-being, which tracks into adulthood. It is therefore important for youth to be facilitated into lifelong active participation in physical activity and sport. Despite the importance of sports injury prevention in youth, no broad scale approaches that work in real-life situations with significant positive effects exist. Sports coaches (SC) and physical educators (PE) experience current approaches exercises as not context specific, time consuming and not contributing to their training goals. This leads to poor uptake, implementation and maintenance of current sports injury prevention exercises. To overcome current barriers, the Move Healthy project develops ICT based video material of routines for and with PE and SC, which supports them to prevent sports injuries in youth. The purpose of this crucial mapping phase is to identify the wishes and needs of the end-users PE and SC regarding injury prevention.

**Methods:**

A mapping procedure was conducted to identify the wishes and needs of PE and SC regarding integration of injury prevention in their daily work. For that, focus groups, with PE from primary and secondary education and basketball and soccer coaches from 6 EU countries, were held.

**Results:**

A total of 31 PE (primary and secondary school) and 37 sport coaches (basketball and soccer) from 6 EU countries were included. The qualitative results from SC state that injury preventive routines should focus on: sport performance, good quality of movement, dynamic combinations of existing exercises and it should be fun and challenging. PE tend to focus more on: motivation aspects, how to overcome barriers and implementation aspects. For both groups, a clear and specific explanation about the why, what and how regarding injury prevention based on principles of motor learning should be included in the approach.

**Conclusions:**

The results from the mapping phase will lead to guidelines, statements regarding educational concepts, content and design criteria for video material on the ICT based support platform for SC and PE.

**Acknowledgements**

This project is financially co-funded by the Erasmus+ Sport program from the European Union.

